# Using Reclaimed Cement Concrete in Pavement Base Mixes with Foamed Bitumen Produced in Cold Recycling Technology

**DOI:** 10.3390/ma15155175

**Published:** 2022-07-26

**Authors:** Justyna Stępień, Krzysztof Maciejewski

**Affiliations:** Department of Transportation Engineering, Faculty of Civil Engineering and Architecture, Kielce University of Technology, Aleja Tysiąclecia Państwa Polskiego 7, 25-314 Kielce, Poland; kmaciejewski@tu.kielce.pl

**Keywords:** reclaimed cement concrete, reclaimed asphalt pavement, reclaimed aggregate, secondary setting, road base mixes, cold recycling, foamed bitumen

## Abstract

The paper presents the results of exploratory research on the use of reclaimed cement concrete in cold-recycled mixes with foamed bitumen. Because reclaimed cement concrete, unlike natural aggregates, is expected to have a residue of the non-hydrated cement covering the aggregate grains, which may result in a secondary cementation process after its application in a road base, this avenue was explored by tracking the time evolution of the compressive strength of the final material. The tests were performed using two mixtures, i.e., a reference mixture and a mixture containing 25% reclaimed cement concrete. The mixtures containing reclaimed cement concrete were characterized by increased uniaxial compressive strengths after each curing period (3, 4, 7, 14 and 28 days)—by 11.5 kPa on average and e.g., 498 kPa vs. 506 kPa after 28 days. The obtained differences between the mixtures were not found to be statistically significant. The small effects of the incorporation of reclaimed cement concrete were attributed to the time passed typically between the demolition and new pavement construction and to the presence of a second binding material—bitumen.

## 1. Introduction

### 1.1. Use of Recycled Materials in Pavement’s Upper Structural Layers

Recycling techniques are now widely used in road construction and they most prominently include producing top asphalt pavement courses with reclaimed asphalt pavement (RAP) and deep cold recycling techniques, enabling road bases to be produced using recycled materials in high quantities, as well as other underlying structural materials. The main purpose of using deep cold recycling technology is to maximize the use of the material obtained from the old worn-out layers of the road surface [1,2,3,4,5]. Most of the roads reconstructed with this technology are pavements with flexible or semi-rigid structures [6,7] and the processed structural layers may, in addition to asphalt layers [8], include improved subgrade [9], cement concrete [10] and unbound mixtures. Materials obtained from the above-mentioned layers come in the form of reclaimed asphalt pavement (RAP), reclaimed cement concrete (RCC) and reclaimed aggregates (RA) from unbound mixtures, which all can be used in the composition of a recycled road base layer. It should be borne in mind that the layout of the existing construction layers determines the percentage share of individual components in the mixture of the recycled base course. The most commonly used amount of reclaimed asphalt is in the range of 20–70% depending on the specific construction, climatic or operating conditions of the rehabilitated pavements, enabling the reuse (recycling) of a large amount of other used building materials. These percentages typically obtain high levels of mechanical performance and resistance to moisture. An amount of RAP equal to 20% was used in research by Niazi and Jalili [11] for assessing the effect of Portland cement and hydrated lime on the properties of a mineral-binder mixture with foamed asphalt. It was shown that the addition of 2% of Portland cement to the mixture improved its indirect tensile strength in dry conditions (ITS_dry_) by approx. 70%, and after water conditioning (ITS_wet_) the increase was 250%, significantly improving the water sensitivity (TSR). Iwański and Chomicz-Kowalska [2], when assessing compaction methods, used 50% RAP in the composition of the base mix and obtained ITS_dry_ values exceeding 500 kPa and ITS_wet_ values of over 400 kPa, regardless of the compaction method used. Iwański and Buczyński used approximately 40% to 60% reclaimed asphalt pavement [12,13] and obtained a material with extremely high stiffness (exceeding 10,000 MPa under dynamic loading) with even higher ITS values (above 1.1 MPa in dry state) when high contents of fines were introduced. The influence of the amount of reclaimed asphalt (0%, 50% and 70%) in the composition of the recycled base with foamed asphalt on the change of the complex modulus was presented by Godenzoni et al. [14] who obtained values approx. in the range of 1000–3000 MPa. These results show that even when very high rates of recycled materials are used in these types of mixtures, adequate mechanical performance can be achieved. In the design process, adequate mix gradation, fines content and amounts of hydraulic binder have to be used to provide adequate mechanical performance and moisture resistance and to mitigate over-stiffening of the material. 

Regarding the cold-recycled base mixes with foamed asphalt, in the case of improper graining of the mineral mix in the existing layers, it is required to introduce a new grading aggregate. The use of a new component translates into an increase in the costs of making a recycled foundation. As a result, it is more advantageous to increase the cost of equipment and bring the existing layers to the required grain size by, e.g., grinding or introducing other types of recycled materials. This approach makes it possible to use the materials from the existing construction layers to the maximum extent in the recycled base course. 

The relative proportions of the individual components (reclaimed material, grading material, hydraulic binder, asphalt binder), as well as the type of the reclaimed portion of the mix, determine the strength and deformation properties of MCAS mixtures and the future construction layer of the road surface.

### 1.2. Recycling of Portland Cement Concrete in Pavement Construction

Most of the waste construction materials which can be used again for producing pavement structures are typically sourced from the demolition and reconstruction of old road, railroad and bridge infrastructure. These waste materials are usually referred to as construction and demolition waste (CDW). Concrete waste is also created as a by-product during production in the fabrication plants of building materials, such as ready-mixed concrete or prefabricates [15].

The effects of the development of the construction market are an increased demand for concrete and an excessive amount of construction waste. Current efforts are focused on environmental protection as a result of a demand to lower the energy consumption of production processes. These include: use of recycling technology in many industrial sectors, enforcing rational waste management and using waste from the road construction sector. Concrete used in old pavements and other concrete structures has a high recycling potential. Thanks to the use of reclaimed concrete in construction, the need for new natural resources can be decreased. The use of recycled building materials lowers the costs of mining and transporting new rock materials [16].

Concrete aggregate may have different grain sizes and functional properties depending on the crushing method. The results of research conducted by Pedro et al. in Portugal [17] indicate, however, that the aggregate properties have a greater impact on the strength and deformation parameters of materials made of such aggregate than the crushing method itself. Sabai et al. [18] showed that the parameters of prefabricated elements based on recycled concrete aggregate allow their use in new building structures. However, it was found that prefabricated elements made of recycled concrete aggregate are characterized by lower strength compared to elements produced based on natural aggregate. This may be due to the greater water absorption of recycled aggregate compared to natural aggregates. In the studies of Wagih et al. [19], it was shown that the content of recycled aggregate at the level of 25% does not significantly affect the properties of cement concrete. Concretes with the content of 25–50% of recycled aggregate were characterized by lower strength parameters compared to conventional concretes, but their features allowed the use of the final product in structures. At the same time, high water absorption and high abrasiveness of recycled concrete aggregate have been demonstrated.

All over the world (including in China, the USA and Norway), reclaimed concrete resulting from the crushing process is used as a road foundation in road structures [20,21,22]. Moreover, reclaimed concrete is classified as a material suitable for the construction of auxiliary foundations, main foundations and cut-off layers, as well as a filler in sound-absorbing embankments [23,24,25]. Reclaimed concrete was investigated for its use in asphalt concrete, including hot and warm mix asphalt techniques [26,27,28,29].

Reclaimed concrete material, in contrast to natural aggregate material, is distinguished by the presence of a hydrated cement matrix that remained on the top layer of the aggregate grains, becoming a component of the hardened concrete mix. Therefore, reclaimed concrete may be subject to additional cementation, which is a consequence of the release of unbound pozzolanic particles after crushing of the original material [30]. Residues of pozzolanic particles result in a reduction in the specific density of grains, a greater ability of water to enter the waste material and a reduction in the quality of aggregate contained in the concrete waste [31,32,33]. During the setting of cement concrete, not all of the cement particles are hydrated, hence the aggregate from the recycling of cement concrete undergoes secondary setting over time, which causes the physical and mechanical parameters of the base layer to change over time. The confirmation of this effect of secondary cementation in the layer of aggregate from recycled concrete was shown [34] both in laboratory tests and in the example of the value of the secondary modulus of deformation of the foundation layer on the road surface.

Based on the information presented above, an investigation was conducted to evaluate the effects of reclaimed cement concrete on the uniaxial compressive strength of deep-cold recycled mixture with foamed bitumen. The study involved evaluating the time evolution of the unconfined compressive strength of a reclaimed concrete-bearing and a reference mixture and included the use of a fine fraction of the reclaimed cement concrete material. The evaluation of uniaxial compressive strength was selected as the most basic material strength property with uniform stress and strain fields. The tests were conducted without any additional use of hydraulic binder to expose the evaluated effects. The objective was to conduct an initial assessment of the possibility and feasibility of utilizing this material, as well as to evaluate the potential presence of secondary setting in the reclaimed cement concrete material.

## 2. Materials and Methods

### 2.1. Experimental Plan

**Figure 1:**
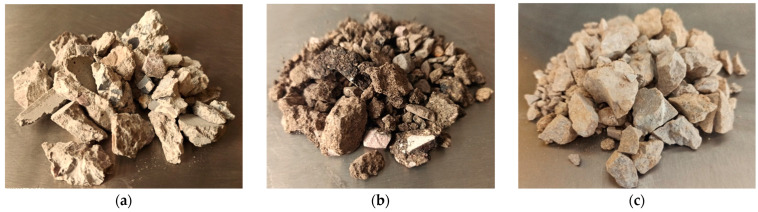
Reclaimed materials comprising the cold-recycled mixtures with foamed bitumen: (**a**) reclaimed cement material; (**b**) reclaimed asphalt pavement (RAP); (**c**) reclaimed aggregate (RA).

In order to evaluate the use of reclaimed cement concrete (RCC) in cold-recycled mixtures with foamed bitumen, an experimental plan was set up to investigate the unconfined compressive strength of such mixtures containing RCC material. The study involved comparing a reference mixture (*REF-Mix*) with a mixture containing 25% of the reclaimed cement concrete material (*RCC-Mix*). To formulate the investigated mixtures, a number of materials were selected (mostly recycled and reclaimed):

- reclaimed cement concrete (RCC) from reclaimed cement concrete road slabs acc. EN 13242:2002+A1:2007 used in exchange with a virgin 0/4 dolomite aggregate,
- reclaimed asphalt pavement (RAP) acc. EN 13108-8:2016,
- reclaimed aggregate (RA) acc. EN 13242:2002+A1:2007, shown in Figure 1.

#### 2.1.1. Recycled Materials Used in the Mixtures

##### Reclaimed Cement Concrete (RCC)

Recycled and reclaimed concrete materials are produced from the demolition of existing concrete structures. The methods for obtaining these materials include demolition or milling of slabs, beam walls of buildings, parts of engineering structures and pavements comprising hydraulically bound layers. This reclaimed material can be used as a construction aggregate or an anthropogenic subgrade [35,36]. 

The investigation utilized reclaimed concrete material from used concrete road slabs. The original reclaimed cement material (RCM) was prepared by 2-mm sieve screening which produced the reclaimed cement concrete (RCC) to be used in the *RCC-Mix.* The grading of the sieved RCC material was made comparable to that of the natural aggregate grading improving material used in the *REF-Mix*. Additionally, this increased the share of the fraction responsible for the presumed formation of secondary cementation, i.e., <0.6 mm [37]. 

**Figure 2:**
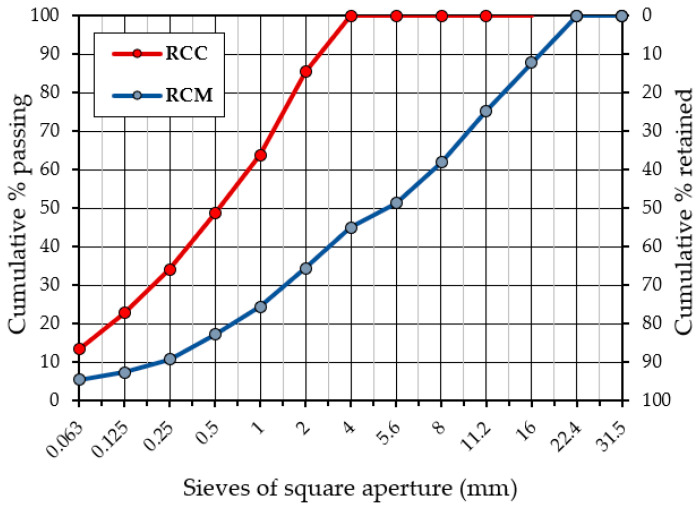
Particle size distribution of reclaimed cement material (RCM) and reclaimed cement concrete (RCC).

The results of the tests for determining the granulometric composition of the original reclaimed concrete and the sieved material prepared from reclaimed concrete according to [38] are presented collectively in Figure 2 and are summarized in Table 1. Table 2 presents a summary of the obtained results for the geometric and physical properties of reclaimed cement material (RCM).

The physical and mechanical properties of reclaimed cement material (RCM) are similar to the physical and mechanical properties of new continuously graded aggregates produced in mineral raw materials mines. Therefore, it can be concluded that it is possible to use such a recycled material in the composition of the mineral-cement mixture with foamed bitumen (MCAS).

**Figure 3:**
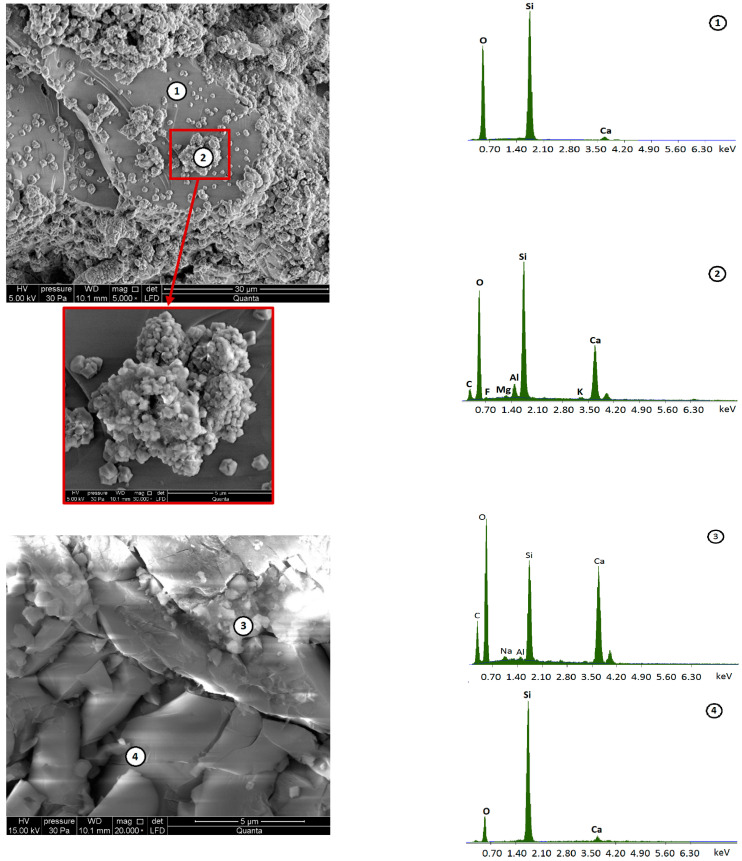
Scanning electron microscope image of the reclaimed cement concrete (RCC) using the Quanta Feg 250 SEM and EDS spectrum at measuring points 1–4.

The chemical composition of the reclaimed cement concrete (RCC) used in the experiments is given in Table 3. EDX (energy-dispersive X-ray) spectroscopy was used to validate the composition of the RCC. The samples for analysis were prepared by covering them with a thin layer of conductive by sputtering with gold (Au). A microstructural analysis of the reclaimed material followed, performed in four distinct selected areas of the investigated sample as shown in Figure 3.

Structural analysis of reclaimed cement concrete material based on scanning electron micrographs showed the presence of cement hydration products, which were accompanied by the formation of calcium hydroxide and a stable structure of hydrated calcium silicates (CSH). The microstructure of the hydrating cement slurry is enlarged for Item 2 (Figure 3). The analysis of the chemical composition also shows the presence of aluminum, magnesium and sodium compounds to a lesser extent at points 2 and 3. There are also visible pores, not filled with CSH gel. The chemical composition identified in the places of grain occurrence (points 1 and 4) shows the presence of mainly silicon and, to a lesser degree, calcium.

##### Reclaimed Asphalt Pavement (RAP)

**Figure 4:**
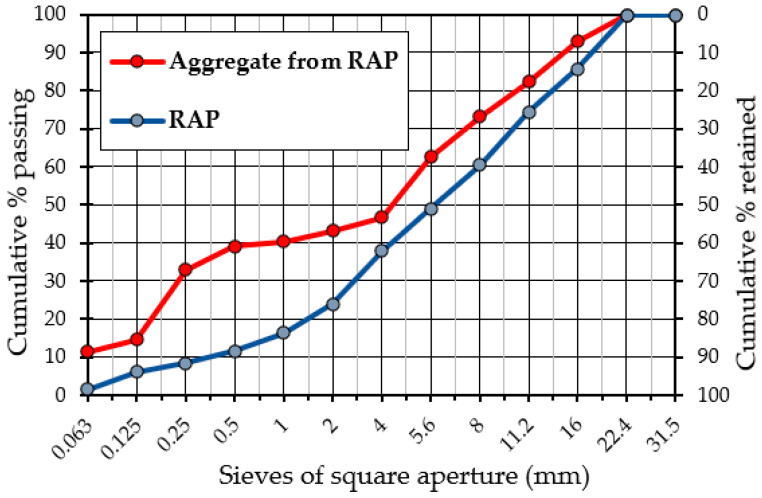
Particle size distribution of reclaimed asphalt pavement (RAP) used in mixture and aggregate from RAP (after extraction).

A major component comprising the investigated mixtures was reclaimed asphalt obtained from the milling of existing asphalt courses (wearing and binding layer), which contained 5.6% of bituminous binder as determined in the extraction test in accordance with EN 12697-1:2012. The evaluation of the particle size distribution of reclaimed asphalt and recovered aggregate (after solvent extraction of the binder) determined in accordance with [38] are shown in Figure 4 and are summarized in Table 4.

Knowing the maximum particle size of reclaimed asphalt mixture (U) and the aggregate grain size after extraction of the binder, the tested reclaimed asphalt, in accordance with the 13108-8: 2016 standard, was designated as 16 RA 0/8, i.e., reclaimed asphalt material with aggregate size of 8 mm and asphalt particles of a maximum size of 16 mm.

Basic tests were carried out based on the EN 13108-8: 2016 standard for the asphalt extracted from the RAP. The results of the analysis are presented in Table 5.

Based on the basic tests of asphalt recovered from reclaimed asphalt, it can be concluded that the binder in the RAP, in terms of penetration, can be classified as 50/70 paving grade bitumen. Based on the results of penetration and softening point determinations, the value of the penetration index was determined (PN-EN 12591: 2009). The calculated penetration index was set at IP = −0.1. The obtained result of the average elastic recovery excludes the possibility of classifying the recovered asphalt as a modified binder.

**Figure 5:**
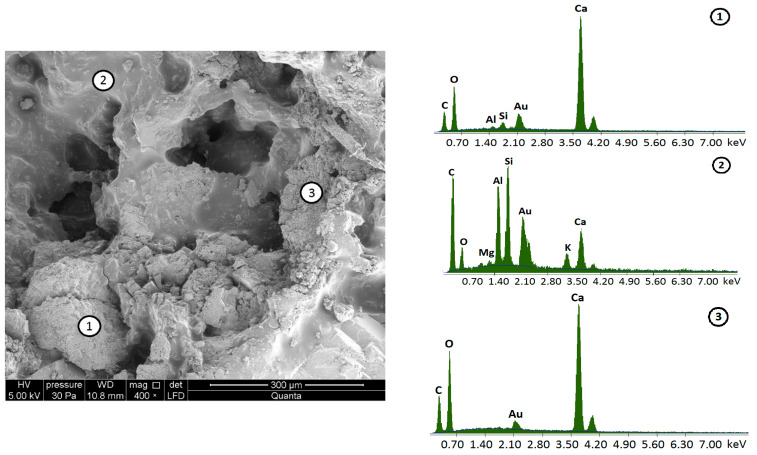
Scanning electron microscope image of the reclaimed asphalt pavement (RAP) using the Quanta Feg 250 SEM and EDS spectrum at measuring points 1–3.

The chemical composition of the RAP used in the experiments is given in Table 6 and its microscopic image is depicted in Figure 5. EDX spectroscopy was used to validate the composition of the RAP. 

Scanning microscope studies revealed the complex structural nature of the material of RAP samples. There are several elements of internal structure with a different chemical composition, defined for example at points 1–3 (Figure 5). Asphalt mastic appears as smooth, amorphous coatings, seen at point 2, with a chemical composition indicating both the presence of asphalt (hydrocarbon compounds) and fine aggregate fractions mainly derived from sedimentary rocks. The analysis revealed the presence of, among others, elements of carbon, silicon, aluminum and calcium. The components of coarse aggregate grains are shown in Items 1 and 3, where the predominant share of calcium compounds was found. There are also visible spaces that are not filled with binding material.

##### Reclaimed Aggregate (RA)

Natural reclaimed aggregate produced by removing an existing road pavement base course was used in this study. This type of material is defined by the EN 13242 standard as an aggregate resulting from the processing of inorganic or mineral material previously used in construction [39]. 

Natural reclaimed aggregates are favored for use in the construction of new infrastructure as it decreases the environmental impact of such endeavors. Utilization of this type of material is particularly well suited to the construction of roads, pavements, bicycle paths, squares and car parks, as well as for levelling roads that do not have bituminous pavements [40]. In many cases, incorporation of reclaimed aggregates in such projects requires additional crushing, sieving and sorting before they can be re-used for the construction of a new road or reconstruction of the existing ones. However, when cold recycling is considered, reclaimed aggregates can often be used in place, without any additional processing. 

Natural reclaimed aggregate utilized in the present study was obtained from an existing unbound road base. The material, which was designated as a continuously graded 0/22 mm size aggregate, was incorporated in both the reference *REF-Mix* and the recycled *RCC-Mix*.

**Figure 6:**
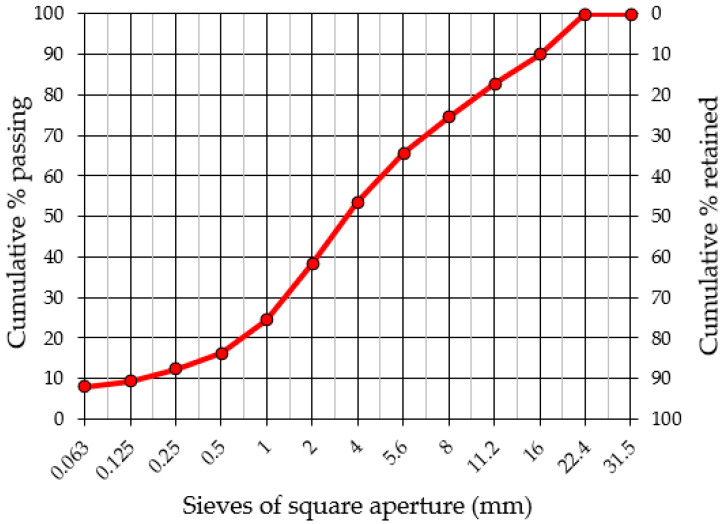
Particle size distribution of reclaimed aggregate (RA) used in the mixture.

The results of the tests for determining the granulometric composition of the reclaimed aggregate according to [38] are shown in Figure 6 and are summarized in Table 7. Table 8 presents a summary of the geometric and physical characteristics.

#### 2.1.2. New Materials Used in the Mixtures

##### Virgin Aggregate (VA)

The reference *REF-Mix* mixture incorporated a 0/4 sized fraction of virgin aggregates in its original design in order to improve its grading. A dolomite aggregate was selected for this purpose because of its wide availability in the region. The utilized dolomite material is typically used in asphalt concrete mixtures and cement concrete formulations and therefore meets all the typical requirements for these kinds of usage. 

**Figure 7:**
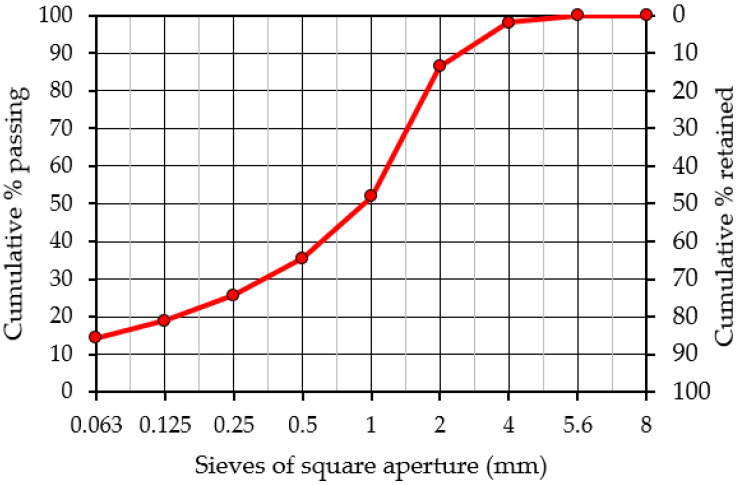
Particle size distribution of the virgin aggregate (VA) used in the mixture.

The results of the tests for determining the grain size composition of aggregate (VA) according to [38] are shown in Figure 7 and summarized in Table 9. Table 10 presents a summary of the geometric and physical characteristics.

##### Foamed Bitumen

**Figure 8:**
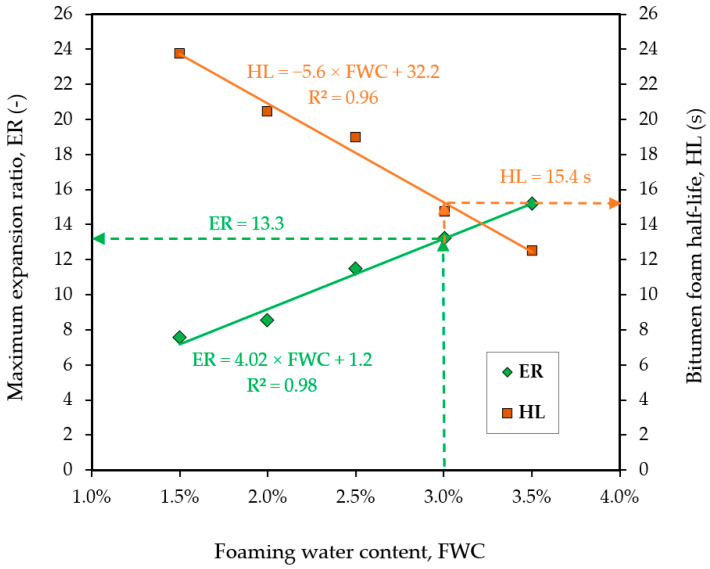
Bitumen foam maximum expansion ratio (ER) and half-life characteristics (HL) of the utilized 50/70 paving grade bitumen.

The bituminous binder used in the investigated mixtures was a 50/70 paving grade bitumen as in [41,42,43] foamed prior to mixing with the mineral material using the WLB10S (Wirtgen, Windhagen, Germany) laboratory foamer. The 50/70 asphalt binder was selected based on the findings of other researchers on the effects of bitumen type on the properties of foamed bitumen mixtures [3]. The properties of the utilized binder are shown in Table 11, while a graphical representation of the determination of foaming water content is presented in Figure 8. 

The physical parameters of the asphalt foam were determined as shown in Figure 8, by introducing a requirement for one of the analyzed features. In this case, an optimal foaming water content FWC = 3.0% (foaming water content) was determined at the values of ER = 13.3 (maximum expansion ratio) and HL = 15.4 s (bitumen foam half-life) as read from the graph.

### 2.2. The Mix Design of Recycled Mixtures

**Figure 9:**
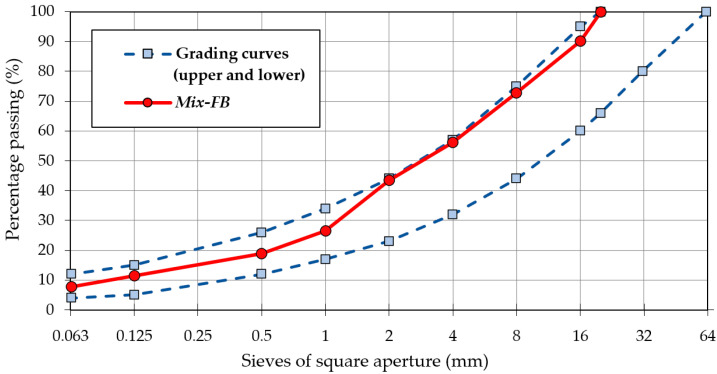
Particle size distribution of the mineral mixtures with boundary curves according to recommendations [44].

The mix design of the recycled mixtures includes the determination of the particle size distribution of the mixture and the contents of binding materials, which typically include foamed asphalt binder and Portland cement, which is required to obtain the necessary moisture and frost damage resistance of cold-recycled mixtures [2,13,44]. However, given the aim of this study, incorporation of fresh Portland cement or any other kind of hydraulic binder would conceal the potential effects of secondary setting in the reclaimed cement concrete material. Therefore, no additional hydraulic binder was used in this study. The mineral composition of the investigated mixtures is shown in Table 12, while their designed particle size distribution is shown in Figure 9. The materials used in the study conformed to the requirements provided in the respective guidelines [36].

Figure 9 shows the requirements regarding particle size distribution regarding cold-recycled mixtures. The designed investigated mixtures conformed to the grading area and both foamed mixtures, *REF-Mix* and *RCC-Mix*, had the same particle size distributions. Both mixtures were designed with the same total asphalt binder content of 5.5%. The asphalt binder comprised in the RAP amounted to 2% of the final mixture and the added foamed bitumen amounted to 3%. The amount of the asphalt binder was in accordance with the relevant recommendations (not exceeding 6%) [44,45].

### 2.3. Optimum Moisture Content (OMC)

**Figure 10:**
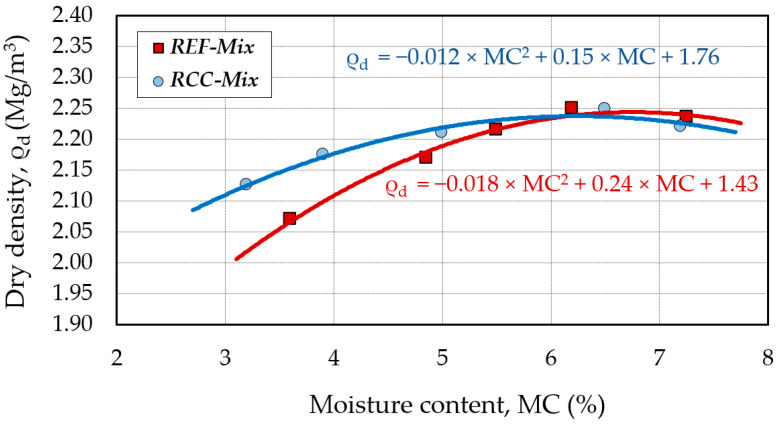
The relationship between moisture content (MC) and dry density (ρ_d_) for the tested mixtures.

The optimum moisture content (OMC) was determined in accordance with the PN EN 13286-2:2010 standard using the Proctor compaction test (large cylinder, method B). The obtained relationship between the moisture content of the mix and its dry density is presented in Figure 10. This relationship permitted establishing the moisture content at the maximum dry density of the mineral mixture which amounted to 6.1%. The mixing moisture content of the recycled mixtures was set at 75% of the OMC.

### 2.4. Experimental Methodology

**Figure 11:**
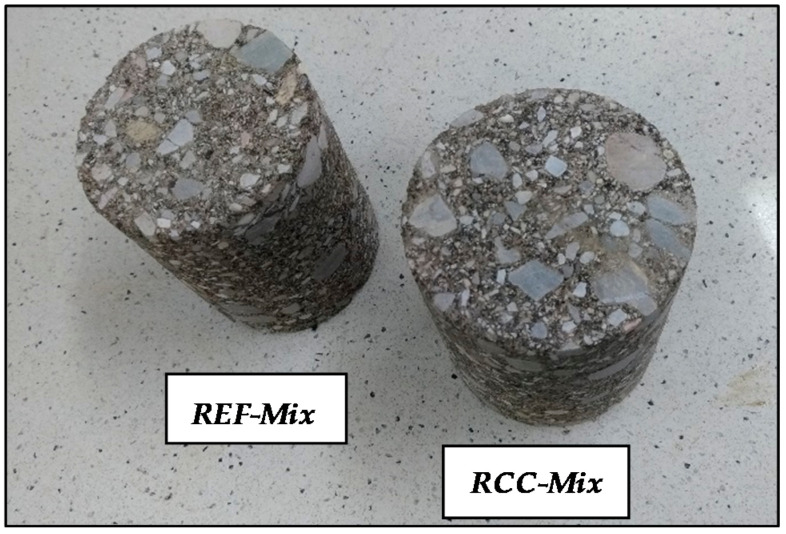
*REF-Mix* and *RCC-Mix* mixture specimens prepared for unconfined compressive strength tests [48].

The experimental tests involved evaluating the unconfined compressive strength (UCS) of the compacted specimens produced from the recycled mixtures at predetermined time intervals of 3, 4, 7, 14 and 28 days of ageing. After compaction, the samples were conditioned for 48 h in the molds and subsequently demolded (Figure 11) and kept in shade on a tabletop. The samples were compacted using the Proctor compactor at 4.6% (75% OMC) according to EN 13286-50 [46]. The evaluation of the unconfined compressive strength was performed to the EN 13286-41 [47] standard at 25 °C.

**Table 12:** Composition of mineral mixtures (mm) and mineral mixtures with foamed bitumen (*Mix-FB*) [48].

Component	Percentage (%)			
	*REF-Mix*		*RCC-Mix*	
	mm	*Mix-FB*	mm	*Mix-FB*
Reclaimed asphalt pavement (RAP)	37	35.7	37	35.7
Reclaimed cement concrete (RCC)	-	-	26	25.1
Reclaimed aggregate (RA)	37	35.7	37	35.7
Virgin aggregate (VA)	26	25.1	-	-
Foamed bitumen 50/70	-	3.5	-	3.5
Total	100	100	100	100

**Figure 12:**
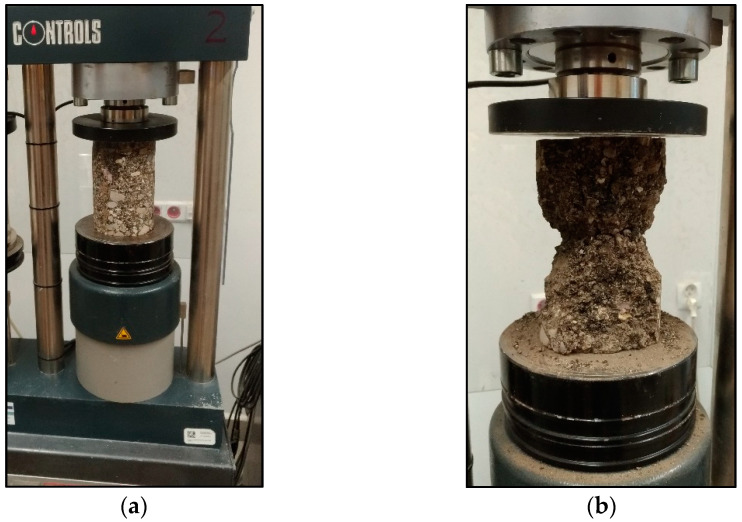
Mixture specimen in UCS testing after reaching ultimate compressive load: (**a**) before the compressive strength test; (**b**) after the compressive strength test [48].

The unconfined compressive strength (UCS) was determined (Figure 12) by measuring the ultimate load to failure of a specimen subjected to a constant loading rate of 142 kPa/s (153 kN/min) [6]. The value of this parameter was determined based on the following relationship [6]:

(1) $$\mathrm{UCS}=\frac{\left(4\times P\right)\times 10000}{\left(\pi \times d^{2}\right)}$$

where:

UCS—unconfined compressive strength (kPa),

P—maximum compressive force (kN),

d—specimen diameter (cm).

## 3. Results and Discussions

**Figure 13:**
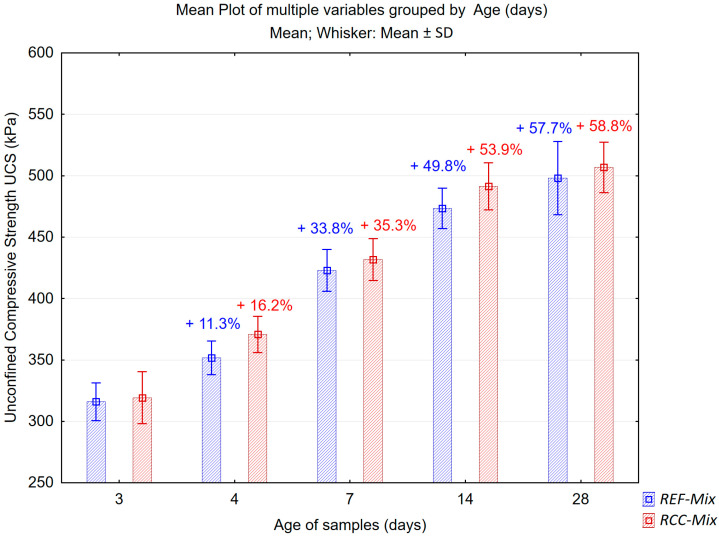
Results of the unconfined compressive strength tests on the samples from the *REF-Mix* and *RCC-Mix* mixtures for the age of samples (age); error bars show standard deviations; increase of unconfined compressive strength shown as a percentage.

**Figure 14:**
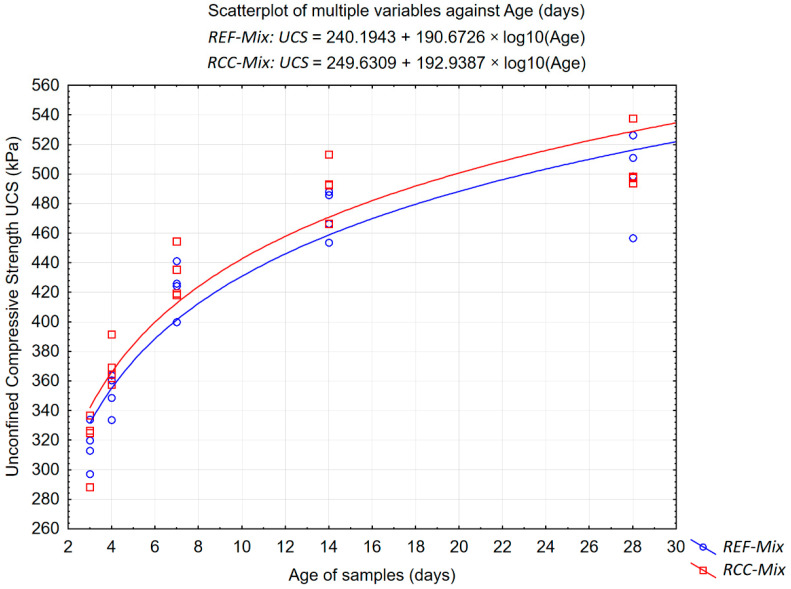
Results of the unconfined compressive strength tests on the samples from the *REF-Mix* and *RCC-Mix* mixtures as a function of specimen age.

The results of the determination of unconfined compressive strength of the investigated recycled mixtures are presented in Figure 13 and Figure 14 in graphical form, while the calculated means, standard deviations, coefficients of variability and relative changes in the measured values are shown in Table 13.

The obtained results of UCS testing have shown that both mixtures exhibit similar strength characteristics throughout the curing period. The reference *REF-Mix,* which did not contain the reclaimed cement concrete aggregates, had a slightly smaller mean value of compressive strength during the whole 28-day period. This difference, which again was not very significant and amounted to 3–9 kPa, could be attributed to the effects of the reclaimed cement concrete in the *RCC-Mix*. The rate at which the compressive strength of the *REF-Mix* and *RCC-Mix* mixtures changed during the curing period decreased over time in a similar manner. This may lead to a conclusion that the processes having the greatest influence on the curing process of both mixtures were similar in nature, i.e., they were not governed primarily by the formation of hydraulic bonds. Nonlinear logarithmic curves were fitted to the data and shown in Figure 14, as these functions are often used to approximate the time evolution of mechanical properties in both cement-bound materials and cold-recycled mixtures [49,50,51]. The results shown in Figure 14 reinforce the above-mentioned observations. The intercept term for the *RCC-Mix* was slightly larger than for the *REF-Mix,* indicating higher initial strength of the mixture with recycled cement concrete. The coefficients of the log functions fitted for both mixtures were similar, indicating a similar increase in strength over time.

To estimate in a more rigorous manner the effects of the RCC material on the unconfined compressive strength of the *RCC-Mix* mixture, a statistical analysis of the obtained results was performed. The results of the initial ANOVA analysis are presented in Table 14 which summarizes the evaluation of the following factors and their effects on the unconfined compressive strength:

- mixture type (*REF-Mix*, *RCC-Mix*),
- sample age (3, 4, 7, 14 days).

The performed analysis of variance reveals that, in the evaluated groups of results, only the age of the investigated samples had a statistically significant effect (α > 0.05) on the mean value of their unconfined compressive strength. It is worth noting, however, that the effect of mixture type (*REF-Mix*, *RCC-Mix*) returned a probability value close to the assumed significance level (*p*-value = 0.066). The significance of the interaction between the mixture type and sample age can, on the other hand, be rejected with high confidence (*p*-value = 0.908). To analyze these initial results in further detail, post hoc Duncan tests were performed to simultaneously compare multiple groups and isolate homogenous groups, between which significant differences could not be proven. The results of this analysis are presented in Table 15.

Analyses of isolated homogeneous groups were in line with the results of the one-way ANOVA analysis showing that for each of the considered sample conditioning periods, the differences between the UCS values of the samples from the *REF-Mix* and *RCC-Mix* mixtures were too small to be statistically significant. The analysis has revealed five distinct groups of mixtures and ageing times which means that the UCS values were statistically similar. The samples produced from the *REF-Mix* and *RCC-Mix* tested at 3, 4 and 7 days yielded statistically similar compressive strength values. The UCS values after 14 and 28 days of conditioning showed even more similarities. The mean UCS values of the samples of the mixtures aged for 14 and 28 days have overlapped in terms of their similarity. This resulted in 14-day *REF-Mix*, *RCC-Mix* and 28-day *REF-Mix* samples being assigned to one homogenous group, and 14-day *REF-Mix* was coupled together with 28-day *REF-Mix* and *RCC-Mix* samples. This result was due to a slow increase in the UCS strength after 14 days of curing and the slight differences between the mixtures’ mean UCS values.

Additional tests were performed to evaluate the *REF-Mix* and *RCC-Mix* separately at different ages (Table 16). The results of these tests have shown that the differences between the unconfined compressive strengths of these mixtures were too small to be proven statistically significant.

## 4. Conclusions

The present study considered using reclaimed cement concrete in cold mixtures with foamed bitumen and has shown a satisfactory performance of the *RCC-Mix* containing recycled cement material.

The preliminary tests performed on the constituent materials have shown their compliance with the adequate requirements, permitting their utilization in recycled foamed mixtures. In particular, the scanning microscope revealed the complex microstructure of the reclaimed materials—the reclaimed cement concrete and reclaimed asphalt pavement. 

The evaluation of the mechanical performance of the produced cold-recycled mixtures with foamed bitumen has shown that the mixtures have increased their unconfined compressive strength in the course of the 28-day curing period. It was also found that the mixture containing the reclaimed cement concrete material experienced a slightly higher increase in strength, although the observed differences were not statistically significant. This observation may be explained by two mechanisms. Firstly, the fine non-hydrated particles easily react with moisture present in air and from other sources. This, together with the typical delay between the demolition and the producing of the new structure, lessens the effects of secondary setting, unless the reclaimed cement concrete is significantly processed (milled, crushed) during the new construction process. Secondly, the presence of bituminous bonding may have reduced the significance of the secondary cement setting in the *RCC-Mix*. This effect may be beneficial when incorporating RCC material into recycled road bases as it reduces the risk of over-stiffening of the road base, and therefore mitigates the possible formation of cracks in the pavement structure.

Future work in this area should include the evaluation of mixtures with higher reclaimed cement content and the evaluation of cracking and fatigue resistance of such mixtures; longer curing times should also be considered. 

## Figures and Tables

**Table 1:** Grading of reclaimed cement material (RCM) and reclaimed cement concrete (RCC).

Sieve Aperture Size (mm)	Percentage of Material Retained 100 × R_i_/M_1_ (% by Mass)	
	RCM	RCC
31.5	0	0
22.4	0	0
16.0	12.2	0
11.2	12.5	0
8	13.4	0
5.6	10.6	0
4	6.3	0
2	10.6	14.5
1	9.9	21.6
0.5	7.3	15.2
0.25	6.4	14.5
0.125	3.4	11.3
0.063	1.9	9.5
<0.063	5.5	13.4
sum	100	100

**Table 2:** Geometric and physical properties of reclaimed cement material (RCM) acc. EN 13242:2002+A1:2007.

Property	Test Method	Result	Category
Aggregate	[38]	continuous grading	
Aggregate sizes (d/D)	[38]	0/16	
Grading and tolerance	[38]	grading curve Table 1, Figure 2	G_A_85, GT_A_20
Flakiness index, %	EN 933-3	9	FI_20_
Shape index, %	EN 933-4	6	SI_20_
Percentage of crushed or broken particles, %	EN 933-5	99. 0	C_90/3_
Fines content, %	[38]	5.5	f_7_
Resistance to fragmentation, Los Angeles test method (10/14 mm), %	EN 1097-2	38	LA_40_
Particle density ρ_a_, Mg/m^3^	EN 1097-6	2.280	declared value
Water absorption, %	EN 1097-6	4.8	WA_24declared_
Freeze–thaw resistance, %	EN 1367-1	3.55	F_4_
Constituents of coarse recycled aggregates Rc + Ru + Rg, %	EN 933-11	99	Rcug_90_

**Table 3:** Mass share of elements detected in EDS analysis of the reclaimed cement concrete (RCC) at measuring points 1–4.

Measuring Point	Si	O	Ca	C	F	Mg	Al	K	Na	Total
1	50.52	46.34	3.14	-	-	-	-	-	-	100
2	26.28	39.81	24.04	5.66	0.48	0.36	2.57	0.80	-	100
3	14.40	43.17	30.94	10.45	-	-	0.38	-	0.65	100
4	70.96	22.37	6.67	-	-	-	-	-	-	100

**Table 4:** Grading of RAP used in mixture and aggregate from RAP (after extraction).

Sieve Aperture Size (mm)	Percentage of Material Retained 100 × R_i_/M_1_ (% by Mass)	
	RAP	Aggregate from RAP
31.5	0	0
22.4	0	0
16.0	14.2	7.2
11.2	11.3	10.4
8	14.0	9.3
5.6	11.6	10.6
4	11.0	15.9
2	13.9	3.7
1	7.7	2.6
0.5	4.8	1.1
0.25	3.0	6.4
0.125	2.5	18.2
0.063	4.5	3.3
<0.063	1.5	11.3
sum	100	100

**Table 5:** Test results of asphalt extracted from reclaimed asphalt pavement (RAP).

Property	Penetration in 25 °C	Softening Point (T_R&B_)	Fraass Breaking Point	Elastic Recovery
Test Method	EN 1426:2015	EN 1427:2015	EN 12593:2015	EN 13398:2017
Unit of Measure	0.1 mm	°C	°C	%
Mean	58	53.2	−5	12.5
Valid N	10	4	4	3

**Table 6:** Mass share of elements detected in EDS analysis of the reclaimed asphalt pavement (RAP) at measuring points 1–3.

Measuring Point	C	O	Al	Si	Au	Ca	Mg	K	Total
1	5.54	25.76	0.54	1.45	12.62	54.09	-	-	100
2	27.38	6.11	8.79	12.75	27.40	12.36	0.53	4.69	100
3	7.59	35.89	-	-	5.90	50.63	-	-	100

**Table 7:** Grading of reclaimed aggregate (RA) used in the mixture.

Sieve Aperture Size (mm)	Percentage of Material Retained 100 × R_i_/M_1_ (% by Mass)
31.5	0
22.4	0
16.0	10.0
11.2	7.3
8	8.2
5.6	8.9
4	12.2
2	15.1
1	14.0
0.5	8.0
0.25	4.0
0.125	3.0
0.063	1.3
<0.063	8.0
sum	100

**Table 8:** Geometric and physical properties of reclaimed aggregate (RA) acc. EN 13242:2002+A1:2007.

Property	Test Method	Result	Category
Grading	[38]	grading curve Table 7, Figure 6	G_A_85
Flakiness index, %	EN 933-3	16	FI_20_
Shape index, %	EN 933-4	23	SI_40_
Fines content, %	[38]	8.0	f_9_
Fines quality, g/kg	EN 933-9	3.5	MB_F_10
Resistance to fragmentation, Los Angeles test method (10/14 mm), %	EN 1097-2	35	LA_30_
Particle density ρ_a_, Mg/m^3^	EN 1097-6	2.650	declared value
Water absorption, %	EN 1097-6	1.2	WA_24_2
Freeze–thaw resistance, %	EN 1367-1	0.3	F_1_

**Table 9:** Grading of virgin aggregate (VA) used in the mixture.

Sieve Aperture Size (mm)	Percentage of Material Retained 100 × R_i_/M_1_ (% by Mass)
8	0
5.6	0
4	1.8
2	11.8
1	34.5
0.5	16.3
0.25	9.9
0.125	6.8
0.063	4.6
<0.063	14.3
sum	100

**Table 10:** Geometric and physical properties of the virgin aggregate (VA) acc. EN 13242:2002+A1:2007.

Property	Test Method	Result	Category
Grading	[38]	grading curve Table 9, Figure 7	GF85
Flow time (0.063/2 mm fraction), s	EN 933-6	39	E_CS_38
Fines content, %	[38]	14.3	f_22_
Fines quality, g/kg	EN 933-9	4.5	MB_F_10
Particle density ρ_a_, Mg/m^3^	EN 1097-6	2.71	declared value
Water absorption, %	EN 1097-6	0.7	WA_24_1

**Table 11:** Properties of the 50/70 road bitumen.

Property	Penetration in 25 °C	Softening Point (T_R&B_)	Fraass Breaking Point
Test Method [0.1 mm]	EN 1426:2015	EN 1427:2015	EN 12593:2015
Unit of Measure	0.1 mm	°C	°C
Mean ± SD	59.9 ± 2.4	48.6 ± 0.4	−16.3 ± 0.6
Valid N	10	4	4

**Table 13:** UCS test results of the samples from *REF-Mix* and *RCC-Mix* mixtures (mean—mean value, SD—standard deviation of the sample, CoV—coefficient of variation, **Δ**—increase of unconfined compressive strength) [48].

Age of Samples (Days)	Mixture Type							
	*REF-Mix*				*RCC-Mix*			
	Mean (kPa)	SD	CoV (%)	Δ (%)	Mean (kPa)	SD	CoV (%)	Δ (%)
3	315.99	15.36	4.9	-	319.14	21.18	6.6	-
4	351.79	13.66	3.9	11.3	370.74	14.71	4.0	16.2
7	422.92	17.10	4.0	33.8	431.81	17.01	3.9	35.3
14	473.48	16.35	3.5	49.8	491.30	19.19	3.9	53.9
28	498.18	29.84	6.0	57.7	506.79	20.57	4.1	58.8

**Table 14:** Analysis of variance for the evaluation of the influence of Type of mixture and Age of samples on the unconfined compressive strength value UCS of the samples from *REF-Mix* and *RCC-Mix* with the use of ANOVA analysis.

Effect	df	F	*p*	Evaluation of Significance
Intercept	1	19,335.57	<0.001	effect significant (α = 0.05)
Mixture type	1	3.64	0.066	-
Age of sample	4	136.41	<0.001	effect significant (α = 0.05)
Mixture type × Age of sample	4	0.25	0.908	-

df—degrees of freedom.

**Table 15:** Homogeneous groups isolated based on the results of the Duncan multiple comparison tests; criterion of no significant differences between the mean UCS values in the samples from the *REF-Mix*, *RCC-Mix* mixtures at significance level α = 0.05 [48].

Mixture Type	Age of Sample	UCS (kPa)—Mean Value	Homogenous Groups (Duncan Post Hoc)				
			1	2	3	4	5
*REF-Mix*	3	315.99			****		
*RCC-Mix*	3	319.14			****		
*REF-Mix*	4	351.79				****	
*RCC-Mix*	4	370.74				****	
*REF-Mix*	7	422.92					****
*RCC-Mix*	7	431.81					****
*REF-Mix*	14	473.48	****				
*RCC-Mix*	14	491.30	****	****			
*REF-Mix*	28	498.18	****	****			
*RCC-Mix*	28	506.79		****			

****—indication of homogeneous groups

**Table 16:** Results of separate *t*-test results comparing the *REF-Mix* and *RCC-Mix* at different ages in terms of UCS values.

Group 1 vs. Group 2	Age (Days)	df	Mean Group 1	Mean Group 2	t-Value	*p*
*REF-Mix* vs. *RCC-Mix*	3	6	315.99	319.14	−0.2406	0.818
*REF-Mix* vs. *RCC-Mix*	4	6	351.79	370.74	−1.8878	0.108
*REF-Mix* vs. *RCC-Mix*	7	6	422.92	431.81	−0.7375	0.489
*REF-Mix* vs. *RCC-Mix*	14	6	473.48	491.29	−1.4133	0.208
*REF-Mix* vs. *RCC-Mix*	28	6	498.18	506.79	−0.4751	0.652

## Data Availability

Data available on request.
